# Supplementary figures and images for: Spectrophotometric-Based Assay to Quantify Relative Enzyme-Mediated Degradation of Commercially Available Bioplastics
Source: Polymers (Basel). 2023 May 24;15(11):2439. doi: 10.3390/polym15112439 (PMC10255859; doi:10.3390/polym15112439)

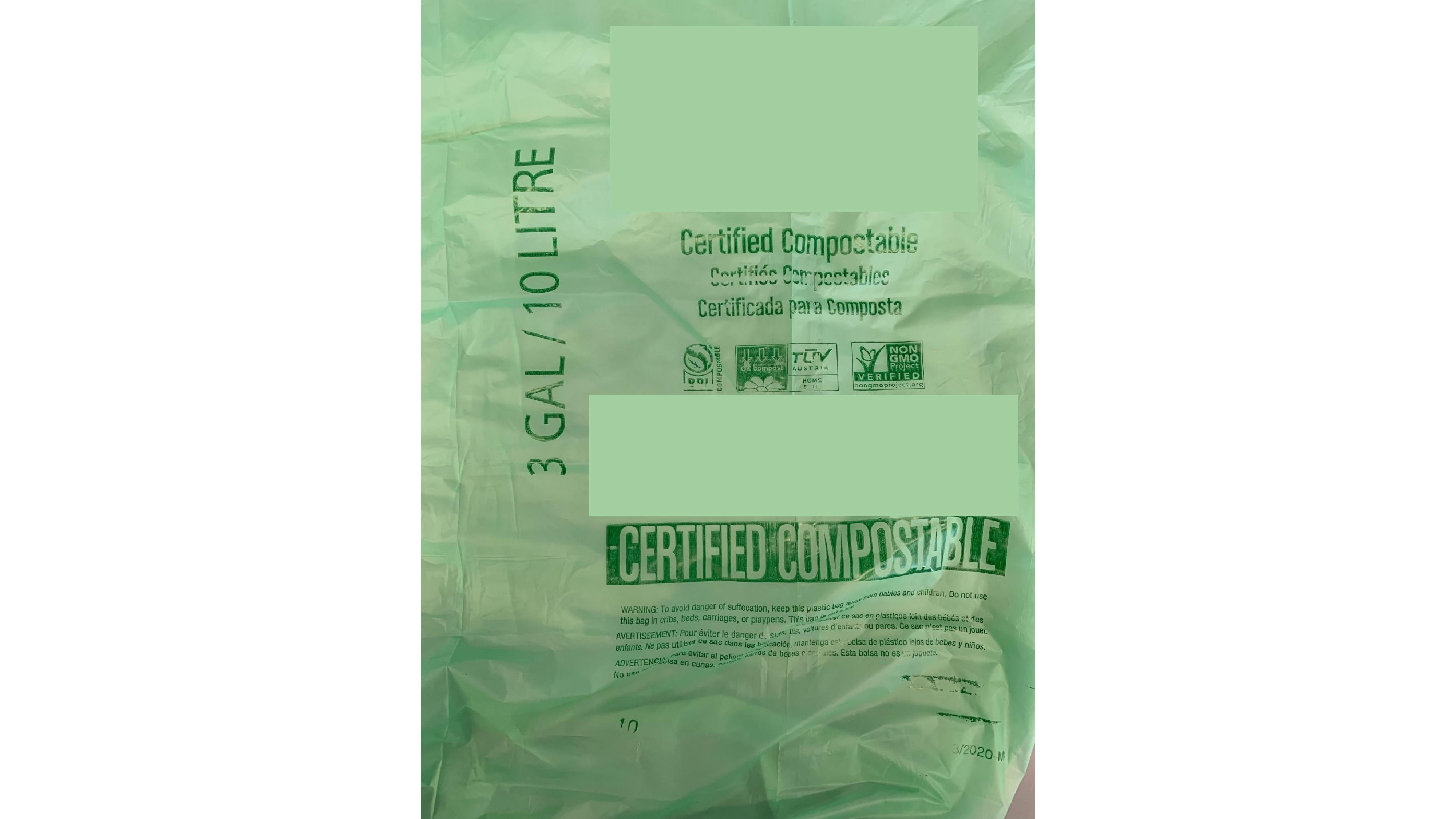

Supplement: Supplementary file 1 [file polymers-15-02439-s001.zip › Supplemental Figure S1.jpg]

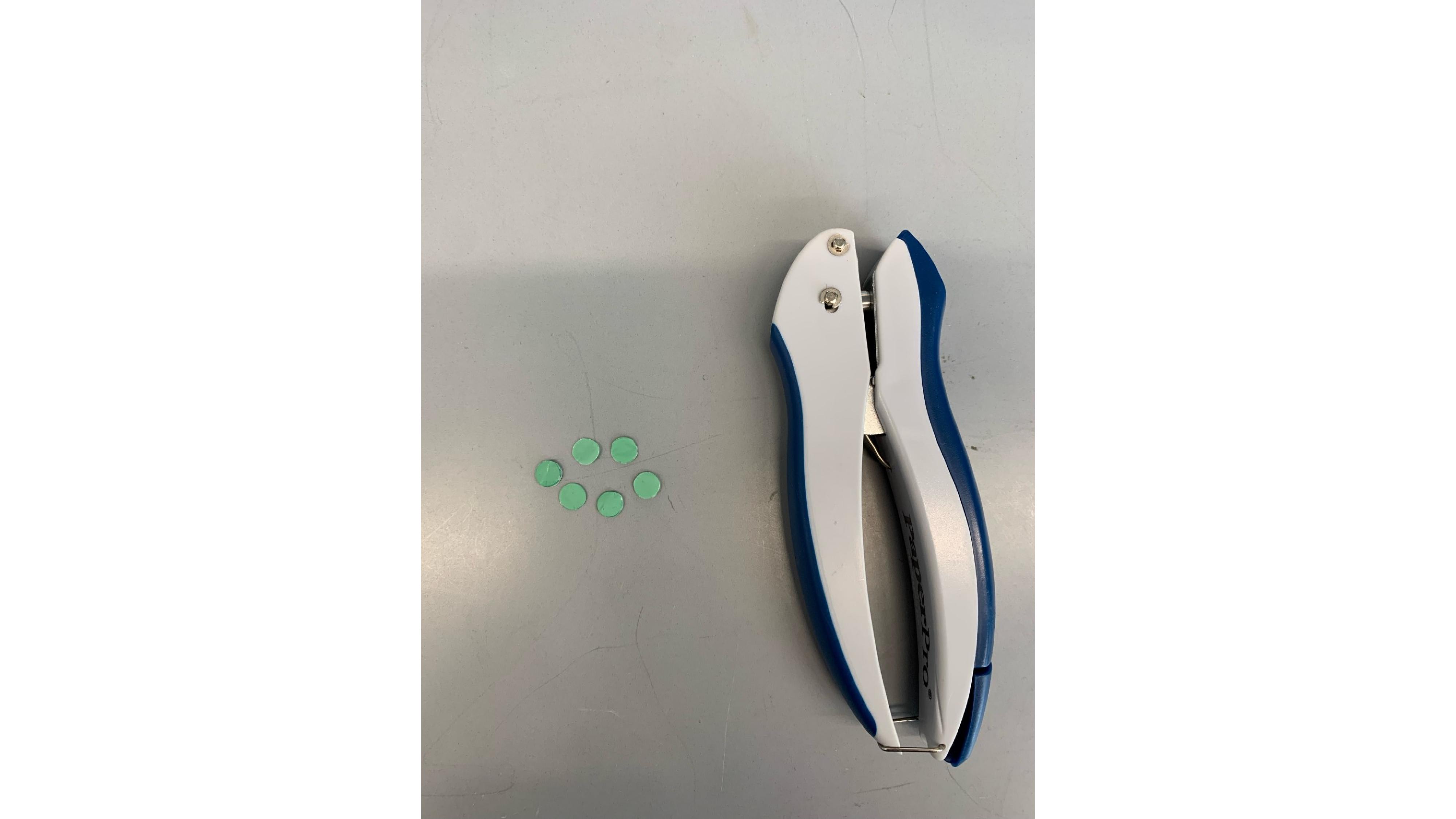

Supplement: Supplementary file 1 [file polymers-15-02439-s001.zip › Supplemental Figure S2.jpg]

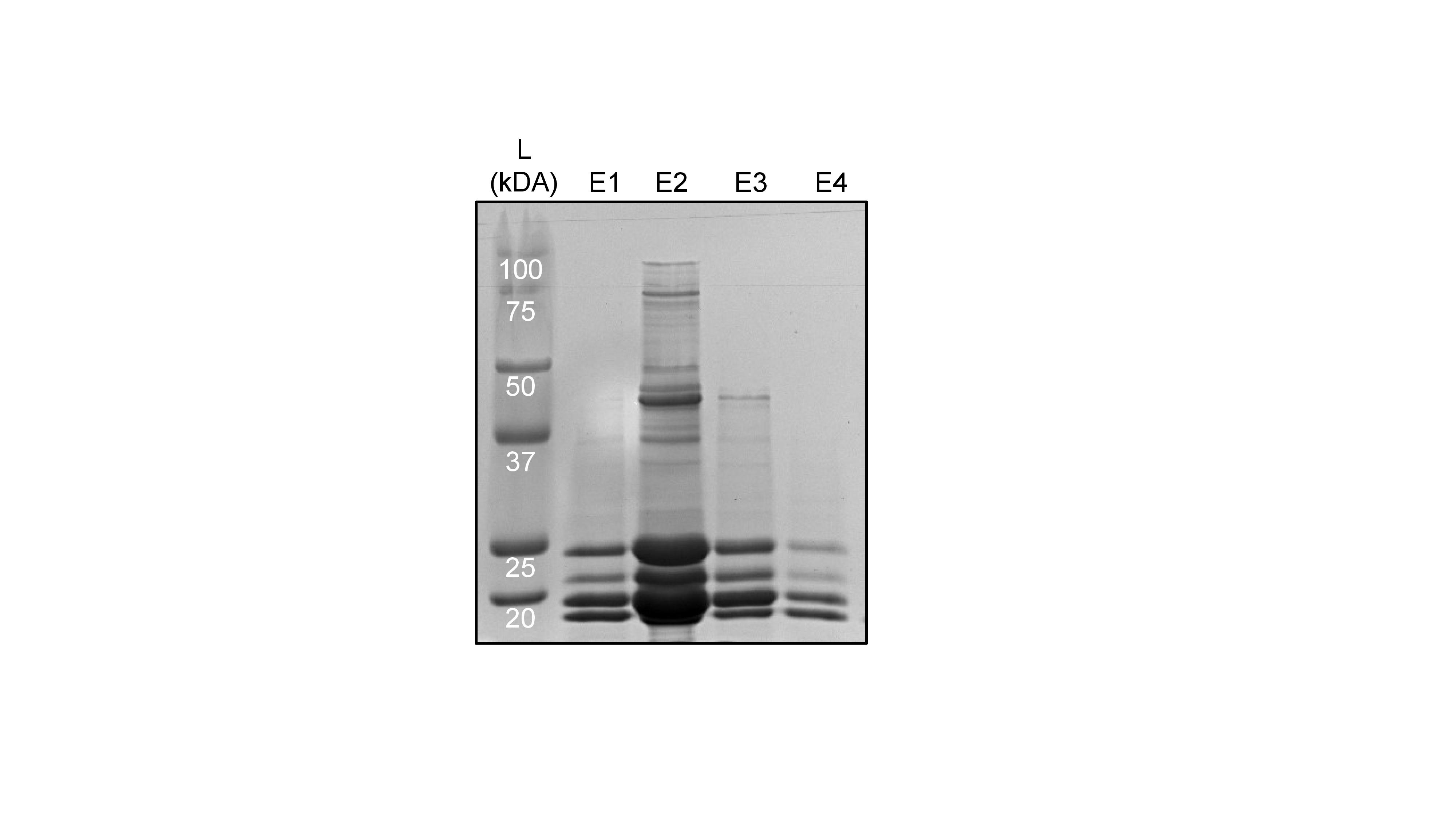

Supplement: Supplementary file 1 [file polymers-15-02439-s001.zip › Supplemental Figure S3.jpg]

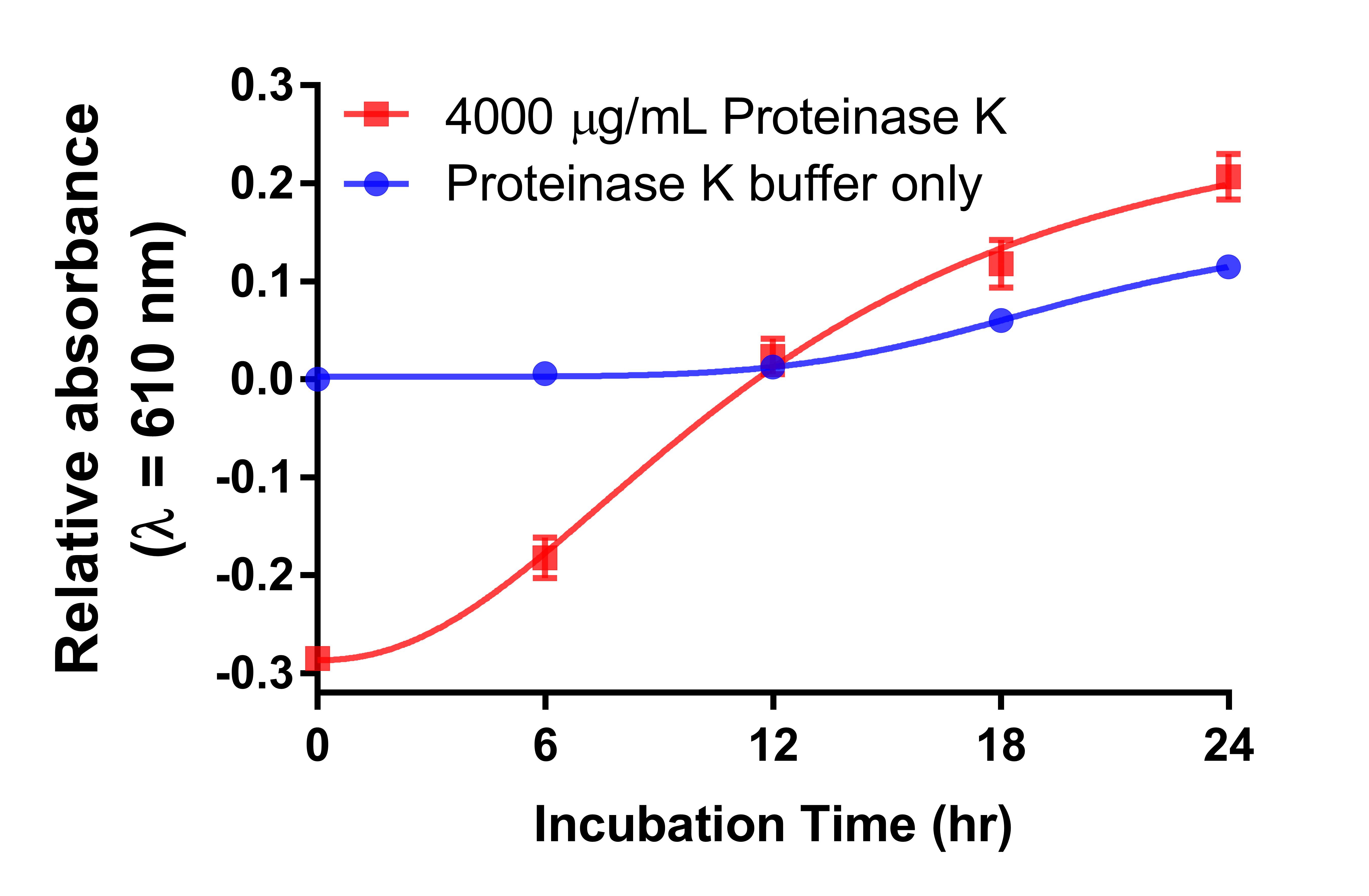

Supplement: Supplementary file 1 [file polymers-15-02439-s001.zip › Supplemental Figure S4.jpg]

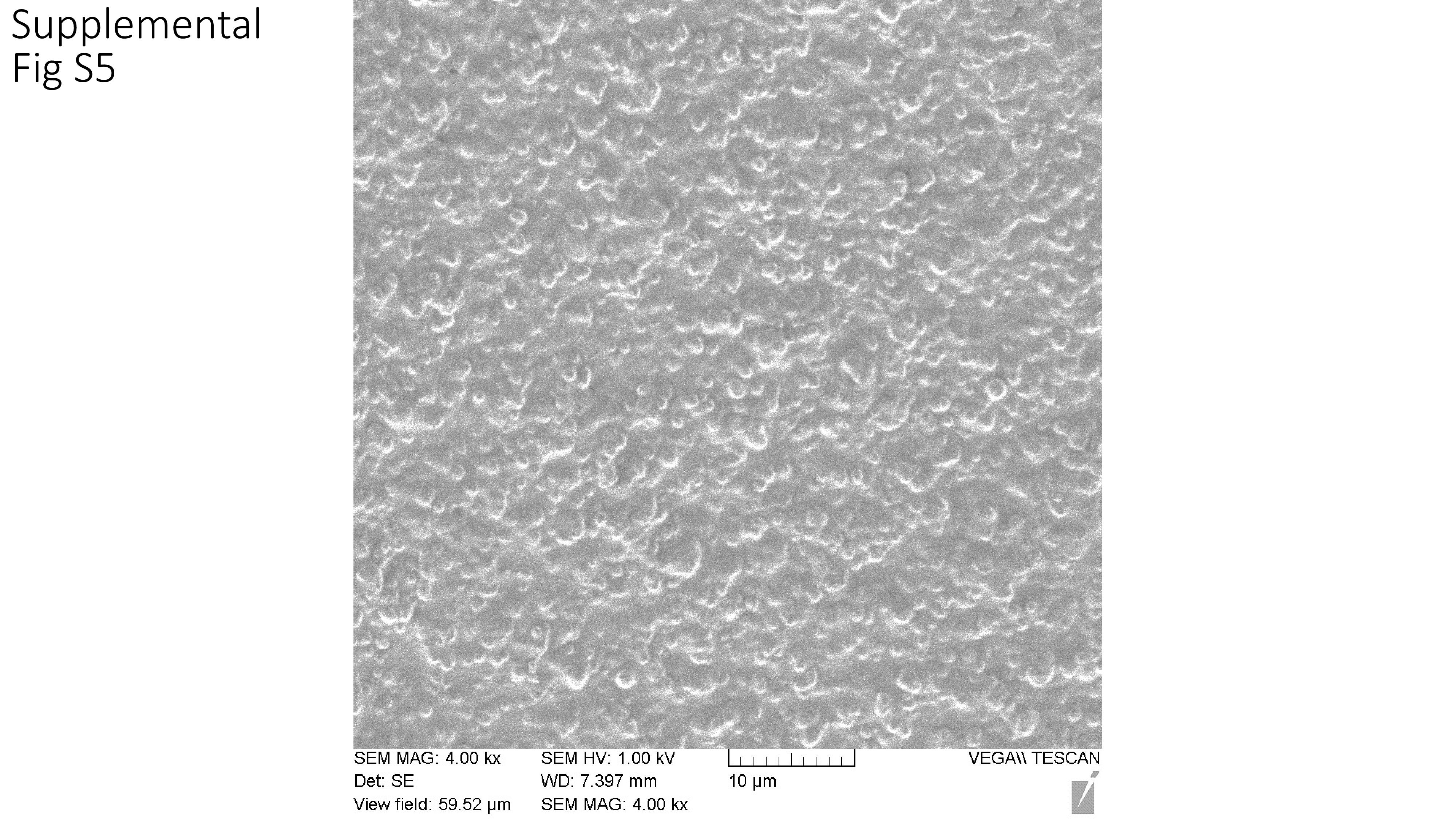

Supplement: Supplementary file 1 [file polymers-15-02439-s001.zip › Supplemental Figure S5.jpg]

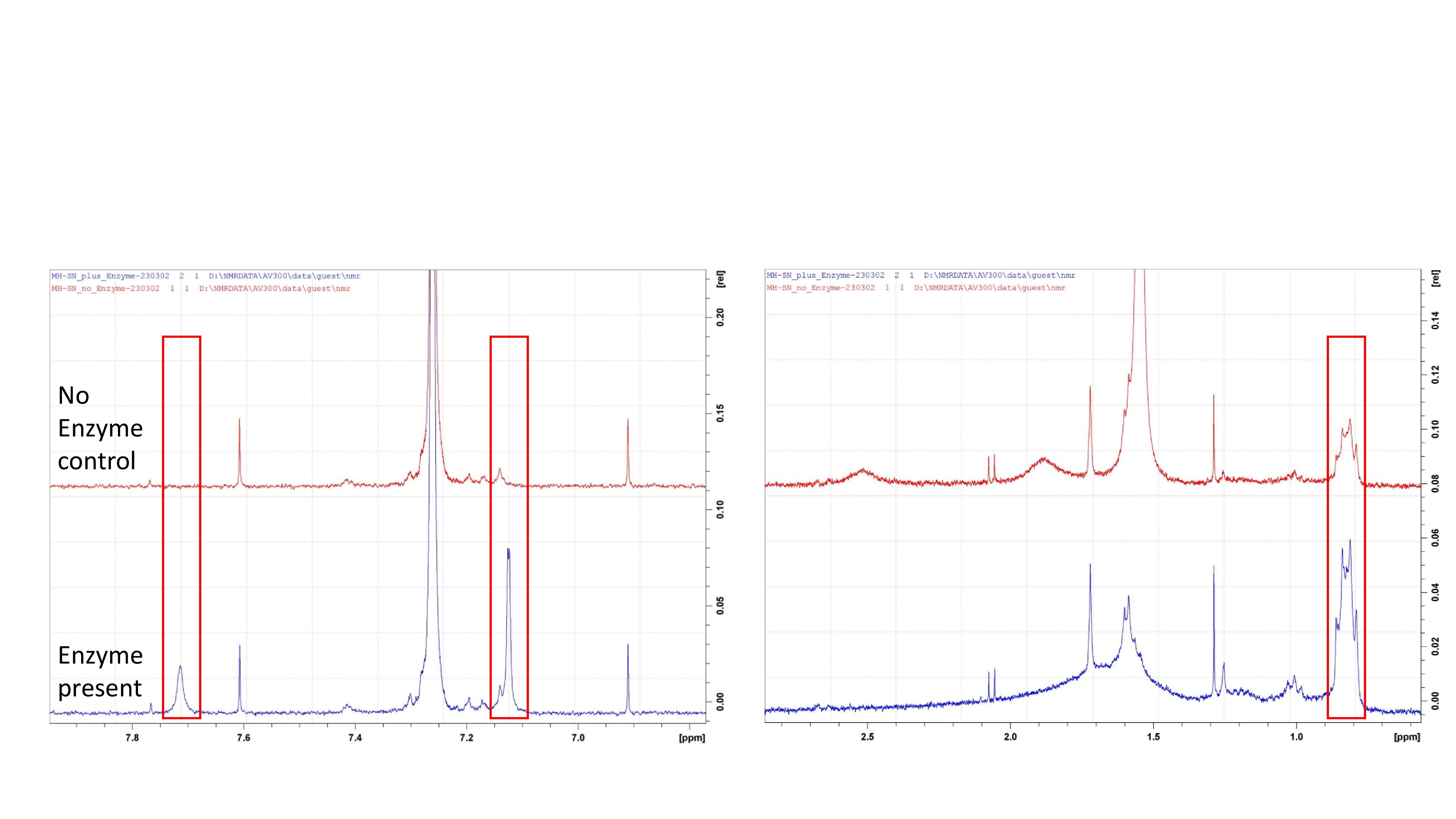

Supplement: Supplementary file 1 [file polymers-15-02439-s001.zip › Supplemental Figure S6.jpg]

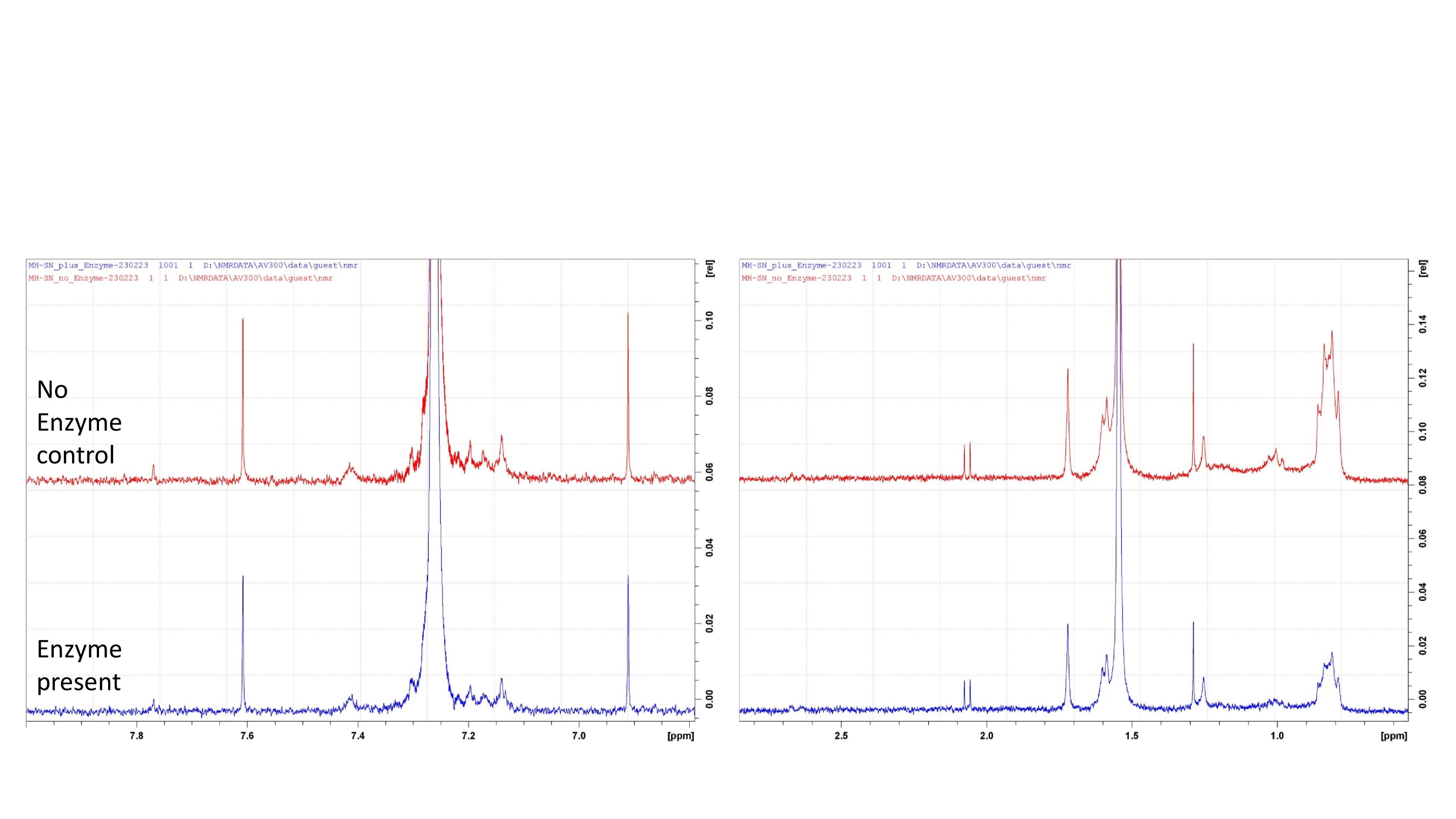

Supplement: Supplementary file 1 [file polymers-15-02439-s001.zip › Supplemental Figure S7.jpg]
